# Views of the Germans on the Operation of Turning

**Published:** 1851-07

**Authors:** Charles West


*Views of the Germans on the Operation of Turning.* By Charles West, M. D.—The doctrine at present taught there with reference to this point is, that turning may be employed with advantage in cases where there exists a moderate degree of pelvic deformity, coupled with not very active pains (which have proved inadequate to drive the head through the pelvic brim), and a dilated state of the os uteri. The younger Osiander has most fully considered this practice, and the objections that have been raised to it, and recommends it as more generally applicable than the majority of his countrymen are disposed to admit, since he is inclined to practise it in many cases as a substitute, not merely for the forceps, but, after they have been unsuccessfully tried, as a means of avoiding the use of the perforator. He denies the danger or difficulty of pushing back the head, and turning the child after the forceps have been ineffectually applied, and states that he has under such circumstances turned with facility. He denies that the difficulty in extracting the head when it comes last is as great as when it presents, and appeals to general experience in proof of his statement. "The reason why the child which could not be brought into the world while its vertex presented, can be delivered after it has been turned, appears to be, that when the head presents, its broader part has to be drawn through a narrow passage without its being held fast, except at its sides; while, on the other hand, after turning, it offers the under part of the face and the neck like the narrow end of a wedge, and thus its smaller part coming first, can be firmly held on either side by the forceps, and if need be, can have additional force applied to move it, by drawing at the lower jaw, and by pressure on the back of the neck and shoulders.—*Med. Gaz.*

